# Supplementary material for: Heat adaptation of phage T7 under an extended genetic code
Source: Virus Evol. 2021 Dec 1;7(2):veab100. doi: 10.1093/ve/veab100 (PMC8923235; doi:10.1093/ve/veab100)
Supplement: veab100_Supp [file veab100_supp.zip › Sequencing_Analysis.zip!/Sequencing_Analysis/S1/evidence/RA_34.html]

BRESEQ :: Evidence


| Read alignment evidence... | | | | | | | | | | | |
| --- | --- | --- | --- | --- | --- | --- | --- | --- | --- | --- | --- |
|  | seq id | position | | ref | new | freq | score (cons/poly) | reads | annotation | genes | product |
| \* | NC\_001604.1 | 36,121 | 0 | G | A | 54.0% | 1070.6 / inf | 5292 | intergenic (–/–) | *–/–* | –/– |
| Reads supporting (aligned to +/- strand):  **ref** base G (1273/1136);  **new** base A (1503/1328);  **total** (2804/2488) | | | | | | | | | | | |
| Fisher's exact test for biased strand distribution *p*-value = 8.68e-01 | | | | | | | | | | | |
| Kolmogorov-Smirnov test that lower quality scores support variant *p*-value = 1.15e-01 | | | | | | | | | | | |

Only 100 of 5327 total aligned reads displayed.  

`GAGGGTCAGATAACAACAATGACTGTACCTTCCACTCCTATGTACATGGTACGACCTTAACACTCAAGCAGGACTATGCAGTAGTTAACAAACACTTCCACGTAGGTCAGGCCGTTGTGGCCACTGATGGTAATATTCAAGGTACTAAGTGGGGAGGTAAATGGCTGGATGCTTACCTACGTG‑ACAGCTTCGTTGCGAAGTCCAAGGCGTGGACTCAGGTGTGGTCTGGTAGTGCTGGCGGTGGGGTAAGTGTGACTGTTTCACAGGATCTCCGCTTCCGCAATATCTGGATTAAGTGTGCCAACAACTCTTGGAACTTCTTCCGTACTGGCCCCGATGGAATCTACTTCATAGCCTCTGATGGTGGATGGTTACGATTCCAAATACACTCCAACGGTCTCGGATTCAAGAATATTGCAGACAGTCGTTCAGTACCTAATGCAATCATGGTGGAGAACGAGTAATTGGTAAATCACAAGGAAAGACGTGTAGTCCACGGATGGACTCTCAAGGAGGTACAAGGTGCTATCATTAGACTTTAACAACGAATTGATTAAGGCTGCTCCAATTGTTGGGACGGGTGTAGCAGATGTTAGTGCT  >  NC_001604.1/35822‑36421`  

                                                                                                                                                                                                                                                                                                            `|`                                                                                                                                                                                                                                                                                                              
     `tCAGATAACAACAATGACTGTACCTTCCACTCCTATGTACATGGTACGACCTTAACACTCAAGCAGGACTATGCAGTAGTTAACAAACACTTCCACGTAGGTCAGGCCGTTGTGGCCACTGATGGTAATATTCAAGGTACTAAGTGGGGAGGTAAATGGCTGGATGCTTACCTACGTG‑ACAGCTTCGTTGCGAAGTCCAAGGCGTGGACTCAGGTGTGGTCTGGTAGTGCTGGCGGTGGGGTAAGTGTGACTGTTTCACAGGATCTCCGCTTCCGCAATATCTGGATTAAGTGTGCcaaca                                                                                                                                                                                                                                                                                                        >  1:336772/1‑301 (MQ=255)`  
         `gtAACAACAATGACTGTACCTTCCACTCCTATGTACATGGTACGACCTTAACACTCAAGCAGGACTATGCAGTAGTTAACAAACACTTCCACGTAGGTCAGGCCGTTGTGGCCACTGATGGTAATATTCAAGGTACTAAGTAGGGAGGTAAATGGCTGGATGCTTACCTACGTG‑ACAGCTTCGTTGCGAAGTCCAAGGCGTGGACTCAGGTGTGGTCTGGTAGTGCTGGCGGTGGGGTAAGTGTGACTGTTTCACAGGATCTCCGCTTCCGCAATATCTGGATTAAGTGTACCAACAActc                                                                                                                                                                                                                                                                                                    <  1:412271/300‑1 (MQ=255)`  
          `tgtataagagacaggTACCTTCCACTCCTATGTACATGGTACGACCTTAACACTCAAGCAGGACTATGCAGTAGTTAACAAACACTTCCACGTAGGTCAGGCCGTTGTGGCCACTGATGGTAATATTCAAGGTACTAAGTGGGGAGGTAAATGGCTGGATGCTTACCTACGTG‑ACAGCTTCGTTGCGAAGTCCAAGGCGTGGACTCAGGTGTGGTCTGGTAGTGCTGGCGGTGGGGTAAGTGTGACTGTTTCACAGGATCTCCGCTTCCGCAATATCTGGATTAAGTGTACCAACAActct                                                                                                                                                                                                                                                                                                   <  1:533803/287‑1 (MQ=255)`  
               `tcggagatgtgtataagagacagtATGTACATGGTACGACCTTAACACTCAAGCAGGACTATGCAGTAGTTAACAAACACTTCCACGTAGGTCAGGCCGTTGTGGCCACTGATGGTAATATTCAAGGTACTAAGTGGGGAGGTAAATGGCTGGATGCTTACCTACGTG‑ACAGCTTCGTTGCGAAGTCCAAGGCGTGGACTCAGGTGTGGTCTGGTAGTGCTGGCGGTGGGGTAAGTGTGACTGTTTCACAGGATCTCCGCTTCCGCAATATCTGGATTAAGTGTGCCAACAACTCTTGGaa                                                                                                                                                                                                                                                                                              <  1:177948/278‑1 (MQ=255)`  
               `acaaTGACTGTACCTTCCAATCCTATGTACATGGTACGACCATAGCACTCAAGCAGGACTATGCAGTAGTTAACAAACACTTCCACGTAGGTCAGGCCGTTGTGGCCACTGATGGTAATATTCAAGGTAGTAAGTGGGGAGGTAAATGGCTGGATGCTTACCTACGTG‑ACAGCTTCGTTGCGAAGTCCAAGGCGTGGACTCAGGTGTGGTCTGGTAGTGCTGGCGGTGGGGTAAGTGTGACTGTTTCACAGGATCTCCGCTTCCGCAATATCTGGATTAAGTGTGCCTACAACTCTTGGaa                                                                                                                                                                                                                                                                                              <  1:506072/301‑1 (MQ=255)`  
                             `ttCCACTCCTATGTACATGGTACGACCTTAACACTCAAGCAGGACTATGCAGTAGTTAACAAACACTTCCACGTAGGTCAGGCCGTTGTGGCCACTGATGGTAATATTCAAGGTACTAAGTGGGGAGGTAAATGGCTGGATGCTTACCTACGTG‑ACAGCTTCGTTGAGAAGTCCAAGGCGTGGACTCAGGTGTGGTCTGGTAGTGCTGGCGGTGGGGTAAGTGTGACTGTTTCACAGGATCTCCGCTTCCGCAATATCTGGATTAAGTGTACCAACAACTCTTGGAACTTCTTCCGTACTg                                                                                                                                                                                                                                                                                >  1:556105/1‑301 (MQ=255)`  
                                  `cTCCTATGTACATGGTACGACCTTAACACTCAAGCAGGACTATGCAGTAGTTAACAAACACTTCCACGTAGGTCAGGCCGTTGTGGCCACTGATGGTAATATTCAAGGTACTAAGTGGGGAGGTAAATGGCTGGATGCTTACCTACGTG‑ACAGATTCGTTGCGAAGTCCAAGGCGTGGACTCAGGTGTGGTCTGGTAGTGCTGGCGGTGGGGTAAGTGTGACTGTTTCACAGGATATCCGCTTCCGCAATATCTTGATTAAGTGTGCCAACAACTCTTGCAACTTCTTCGGTACTGGccct                                                                                                                                                                                                                                                                           >  1:151833/1‑300 (MQ=255)`  
                                    `ccTATGTACATGGTACGACCTTAACACTCAAGTAGGACTATGCAGTAGTTAACAAACACTTCCACGTAGGTCAGGCCGTTGTGGCCACTGATGGTAATATTCAAGGTACTAAGTGGGGAGGTAAATGGCTGGATGCTTACCTACGTG‑ACAGCTTCGTTGCGAAGTCCAAGGCGTGGACTCAGGTGTGGTCTGGTAGTGCTGGCGGTGGGGTAAGTGTGACTGTTTCACAGGATCTCCGCTTCCGCAATATCTGGATTAAGTGTACCAACAACTCTTGGAACTTCTTCCGTACTGGCCCCGa                                                                                                                                                                                                                                                                         >  1:341737/1‑301 (MQ=255)`  
                                    `ccTATGTACATGGTACGACCTTAACACTCAAGCAGGACTATGCAGTAGTTAACAAACACTTCCATGTAGGTCAGGCCGTTGTGGCCACTGATGGTAATATTCAAGGTACTAAGTGGGGAGGTAAATGGCTGGATGCTTACCTACGTG‑ACAGCTTCGTTGCGAAGTCCAAGGCGTGGACTCAGGTGTGGTCTGGTAGTGCTGGCGGTTGGGTAAGTGTGACTGTTTCACAGGATCTCCGCTTCCGCAATATCTGGATTAAGTGTACCAACAACTCTTGGAACTTCTTCCGTACTGGCCCCGa                                                                                                                                                                                                                                                                         >  1:312724/1‑301 (MQ=255)`  
                                    `ccTATGTACATGGTACGACCTTAACACTCAAGCAGGACTATGCAGTAGTTAACAAACACTTCCACGTAGGTCAGGCCGTTGTGGCCACTTATGGTAATATTCAAGGTACTAAGTGGGGAGGTAAATGGCTGGATGCTTACCTACGTG‑ACAGCTTCGTTGCGAAGTCCAAGGCGTGGACTCAGGTGTGGTCTGGTAGTGCTGGCGGTGGGGTAAGTGTGACTGTTTCACAGGATCTCCGCTTCCGCATTATCTGGATTAAGTGTACCAACAACTCTTGGAACTTCTTCCGTACTGGCCCCGa                                                                                                                                                                                                                                                                         >  1:380827/1‑301 (MQ=255)`  
                                     `cTATGTAAATGGGACGAGCTGAAAACGCAAGCAGGACTATGCAGGAGTGAACAAACAATGCAAAGTAGGTCAGGCCGGTGTGGCCACTGAGGGTAAGATGAAAGGTACTAAGTGGGGAGGTAAATGGATGGATGCTGACCTACGTG‑ACAGCGTCGTTGCGAAGTCGAAGGCGTGGACTCAGGTGTGGTCTGGTAGTGCTGGCGGTGGGGTAAGTGTGACTGTTTCACAGGATCTCCGCTTCCGCAATATCTGGATTAAGTGTGCCAACAACTCTTGGAACTTCTTCCGTACTGGCCCCGAt                                                                                                                                                                                                                                                                        <  1:451830/301‑1 (MQ=255)`  
                                      `tATGTACATGGTACGACCTTAACACTCAAGCAGGACTATGCAGTAGTTAACAAACACTTCCACGTAGGTCAGGCCGTTGTGGCCACTGATGGTAATATTCAAGGTACTAAGTGGGGAGGTAAATGGCTGGATGCTTACCTACGTG‑ACAGCTTCGTTGCGAAGTCCAAGGCGTGGACTCAGGTGTGGTCTGGTAGTGCTGGCGGTGGGGTAAGTGTGACTGTTTCACAGGATCTCCGCTTCCGCAATATCTGGATTAAGTGTGCCAACAACTCTTGGAACTTCTTCCATACtctgtctcttt                                                                                                                                                                                                                                                                       >  1:11894/1‑291 (MQ=255)`  
                                      `tATGTACATGGTACGACCTTAACACTCAAGCAGGACTATGCAGTAGTTAACAAACACTTCCACGTAGGTCAGGCCGTTGTGGCCACTGATGGTAATATTCAAGGTACTAAGTGGGGAGGTAAATGGCTGGATGCTTACCTACGTG‑ACAGCTTCGTTGCGAAGTCCAAGGCGTGGACTCAGGTGTGGTCTAGTAGTGCTGGCGGTGGGGTAAGTGTGACTGTTTCACAGGATCTCCGCTTCCGCAATATCTGGATTAAGTGTACCAACAACTCTTGGAACTTCTTCCGTAcctgtctctttt                                                                                                                                                                                                                                                                       >  1:445607/1‑290 (MQ=255)`  
                                       `gagaaagtGGTACGACCTTAACACTCAAGCAGGACTATGCAGTAGTTAACAAACACTTCCACGTAGGTCAGGCCGTTGTGGCCACTGATGGTAATATTCAAGGTACTAAGTGGGGAGGTAAATGGCTGGAAGCTTACCTACGTG‑ACAGCTTCGTTGCGAAGTCCAAGGCGTGGACTCAGGTGTGGTCTGGTAGTGCTGGCGGTGGGGTAAGTGTGACTGTTTCACAGGATCTCCGCTTCCGCAATATCTGGATTAAGTGTACCAACAACTCTTGGAACTTCTTCCGTACTGGCCCCGATgg                                                                                                                                                                                                                                                                      <  1:471438/294‑1 (MQ=255)`  
                                         `gTACATGGTACGACCTTAACACTCAAGCAGGACTATGCAGTAGTTAACAAACACTTCCACGTAGGTCAGGCCGTTGTGGCCACTGATGGTAATATTCAAGGTACTAAGTGGGGAGGTAAATGGCTGGATGCTTACCTACGTG‑ACAGCTTCGTTGCGAAGTCCAAGGCGTGGACTCAGGTGTGGTCTGGTAGTGCTGGCGGTGGGGTAAGTGTGACTGTTTCACAGGATCTCCGCTTCCGCAATATCTGGATTAAGTGTGCCAACAACTCTTGGAACTTCTTCCGTACTGGccctgtctctt                                                                                                                                                                                                                                                                    >  1:625679/1‑293 (MQ=255)`  
                                           `aCATGGTACGACCTTAACACTCAAGCAGGACTATGCAATAGTTAACAAACACTTCCACGTAGGTCAGGCCGTTGTGGCCACTGATGTTAATATTCAAGGTACTAAGTGGGGAGGTAAATGGCTTGATGCTTACCTACGTG‑ACAGCTTCGTCCCGAAGTCCAAGCCGTGGACTCAGGTTTGGTCTGGTAGTGCTGGCGGTGGGTTAAGTGTGACTGTTTCACAGGATCCCCGCTTCCGCAATATCTGGATTAAGTGTGCCAACAACTCTTGGAACTTCTTCCGTACTGGCCCCGATGGAATc                                                                                                                                                                                                                                                                  >  1:135551/1‑301 (MQ=255)`  
                                            `ggctcggagatgtgtataagagacagggACTATGCAGTAGTTAACAAACACTTCCACGTAGGTCAGGCCGTTGTGGCCACTGATGGTAATATTCAAGGTACTAAGTGGGGAGGTAAATGGCTGGATGCTTACCTACGTG‑ACAGCTTCGTTGCGAAGTCCAAGGCGTGGACTCAGGTGTGGTCTGGTAGTGCTGGCGGTGGGGTAAGTGTGACTGTTTCACAGGATCTCCGCTTCCGCAATATCTGGATTAAGTGTGCCAACAACTCTTGGAACTTCTTCCGTACTGGCCCCGATGGAATCt                                                                                                                                                                                                                                                                 <  1:183373/275‑1 (MQ=255)`  
                                            `cATGGTACGACCTTAACACTCAAGCAGGACTATGCAGTAGTTAACAAACACTTCCACGTAGGTCAGGCCGTTGTGGCCACTGATGGTAATATTCAAGGTACTAAGTGGGGAGGTAAATGGCTGGATGCTTACCTACGTG‑ACAGCTTCGTTGCGAAGTCCAAGGCGTGGACTCAGGTGTGGTCTGGTAGTGCTGGCGGTGGGGTAAGTGTGACTGTTTCACAGGATCTCCGCTTCCGCAATATCTGGATTAAGTGTGCCAACAACTCTTGGAACTTCTTCCGTACTGGCCCCGATGGAATCt                                                                                                                                                                                                                                                                 <  1:322729/301‑1 (MQ=255)`  
                                              `tGGTACGACCTTAACACTCAAGCAGGACTATGCAGTAGTTAACAAACACTTCCACGTAGGTCAGGCCGTTGTGGCCACTGATGGTAATATTCAAGGTACTAAGTGGGGAGGTAAATGGCTGGATGCTTACCTACGTG‑ACAGCTTCGTTGCGAAGTCCAAGGCGTGGACTCAGGTGTGGTCTGGTAGTGCTGGCGGTGGGGTAAGTGTGACTGTTTCACAGGATCTCCGCTTCCGCAATATCTGGATTAAGTGTACCAACAACTCTTGGAACTTCTTCCGTACTGGCCCCGATGGAATCTAc                                                                                                                                                                                                                                                               >  1:146387/1‑301 (MQ=255)`  
                                               `ggTACGACCTTAACACTCAAGCAGGACTATGCAGTAGTTAACAAACACTTCCACGTAGGTCAGGCCGTTGTGGCCACTGATGGTAATATTCAAGGTACTAAGTGGGGAGGTAAATGGCTGGATGCTTACCTACGTG‑ACAGCTTCGTTGCGAAGTCCAAGGCGTGGACTCAGGTGTGGTCTGGTAGTGCTGGCGGTGGGGTAAGTGTGACTGTTTCACAGGATCTCCGCTTCCGCAATATCTGGATTAAGTGTACCAACAACTCTTGGAACTTCTTCCGTACTGGCCCCGATGGAATCTACt                                                                                                                                                                                                                                                              >  1:573812/1‑301 (MQ=255)`  
                                                    `gACCTTAACACTCAAGCAGGACTATGCAGTAGTTAACAAACACTTCCACGTAGGTCAGGCCGTTGTGGCCACTGATGGTAATATTCAAGGTACTAAGTGGGGAGGTAAATGGCTGGATGCTTACCTACGTG‑ACAGCTTCGTTGCGAAGTCCAAGGCGTGGACTCAGGTGTGGTCTGGTAGTGCTGGCGGTGGGGTAAGGGTGACTGTTTCACAGGATCTCCGCTTCCGCAATATCTGGATTAAGTGTGCCAACAACTCTTGGAACTTCTTACGTACTGGCCCCTATGGAATCTCATTCATa                                                                                                                                                                                                                                                         >  1:329533/1‑301 (MQ=255)`  
                                                      `ccTTAACACTCAAGCAGGACTATGCAGTAGTTAACAAACACTTCCACGTAGGTCAGGCCGTTGTGGCCACTGATGGTAATATTCAAGGTACTAAGTGGGGAGGTAAATGGCTGGATGCTTACCTACGTG‑ACAGCTTCGTTGCGAAGTCCAAGGCGTGGACTCAGGTGTGGTCTGGTAGTGCTGGCGGTGGGGTAAGTGTGACTGTTTCCCAGGATCTCCGCTTCCGCAATATCTGGATTAAGTTTGCCAACAACTCTTGGAACTTCTTCCGTACTGGCCCCGGTGGAATCTACTTCATAGc                                                                                                                                                                                                                                                       >  1:633656/1‑301 (MQ=255)`  
                                                      `ccTTAACACTCAAGCAGGACTATGCAGTAGTTAACAAACACTTCCACGTAGGTCAGGCCGTTGTGGCCACTGATGGTAATATTCAAGGTACTAAGTGGGGAGGTAAATGGCTGGATGCTTACCTACGTG‑ACAGCTTCGTTGCGAAGTCCAAGGCGTGGACTCAGGTGTGGTCTGGTAGTGCTGGCGGTGGGGTAAGTGTGACTGTTTCACAGGATCTCCGCTTCCGCAATATCTGGATTAAGTGTGCCAACAACTCTTGGAACTTCTTCCGTACTGGCCCCGATGGAATCTACTTCATAGc                                                                                                                                                                                                                                                       >  1:173518/1‑301 (MQ=255)`  
                                                               `tataagagacagaTGCAGTAGTTAACAAACACTTCCACGTAGGTCAGGCCGTTGTGGCCACTGATGGTAATATTCAAGGTACTAAGTGGGGAGGTAAATGGCTGGATGCTTACCTACGTG‑ACAGCTTCGTTGCGAAGTCCAAGGCGTGGACTCAGGTGTGGTCTGGTAGTGCTGGCTGTGGGGTAAGTGTGACTGTTTCACAGGATCTCCGCTTCCGCAATATCTGGATTAAGTGTACCAACAACTCTTGGAACTTCTTCCGTACTGGCCCCGATGGAATCTACTTCACAGCCTCTGAtgg                                                                                                                                                                                                                                              <  1:30648/289‑1 (MQ=255)`  
                                                               `atgtgtataagagacaggtagtTAACAAACACTTCCACGTAGGTCAGGCCGTTGTGGCCACTGATGGTAATATTCAAGGTACTAAGTGGGGAGGTAAATGGCTGGATGCTTACCTACGTG‑ACAGCTTCGTTGCGAAGTCCAAGGCGTGGACTCAGGTGTGGTCTGGTAGTGCTGGCGGTGGGGTAAGTGTGACTGTTTCACAGGATCTCCGCTTCCGCAATATCTGGATTAAGTGTACTAACAACTCTTGGAACTTCTTCCGTACTGGCCCCGATGGAATCTACTTCATAGCCTCTGAtgg                                                                                                                                                                                                                                              <  1:271231/284‑1 (MQ=255)`  
                                                               `aagagacagACTATGCAGTAGTTAACAAACACTTCCACGTAGGTCAGGCCGTTGTGGCCACTGTTGGTAATATTCAAGGTACTAAGTGGGGAGGTAAATGGCTGGATGCTTACCTACGTG‑ACAGCTTCGTTGCGAAGTCCAAGGCGTGGACTCAGGTGTGGTCTGGTAGTGCTGGCGGTGGGGTAAGTGTGACTGTTTCACAGGATCTCCGCTTCCGCAATATCTGGATTAAGTGTACCAACAACTCTTGGAACTTCTTCCGTACTGGCCCCGATGGAATCTACTTCATAGCCTCTGAtgg                                                                                                                                                                                                                                              <  1:221475/293‑1 (MQ=255)`  
                                                                   `gCAGGACTATGCAGTAGTTAACAAACACTTCCACGTAGGTCAGGCCGTTGTGGCCACTGATGGTAATATTCAAGGTACTAAGTGGGGAGGTAAATGGCTGGATGCTTACCTACGTG‑ACAGCTTCGTTGCGAAGTCCAAGGCGTGGACTCAGGTGTGGTCTGGTAGTGCTGGCGGTGGGGTAAGTGTGACTGTTTCACAGGATCTCCGCTTCCGCAATATCTGGATTAAGTGTGCCAACAACTCTTGGAACTTCTTCCGTACTGGCCCCGATGGAATCTACTTCATAGCCTCTGATGGTGGa                                                                                                                                                                                                                                          <  1:550644/301‑1 (MQ=255)`  
                                                                   `gCAGGACTATGCAGTAGTTAACAAACACTGCCACGTAGGTCAGGCCGTTGTGGCCACTGATGGTAATATTCAAGGTACTAAGTGGGGAGGTGAATGGCTGGATGCGTACCTACGTG‑ACAGCTTCGTTGCGAAGTCCAAGGCGTGGACTCAGGTGTGGTCTGGTAGTGCTGGCGGTGGGGTAAGTGTGACTGTTTCACAGGATCTCCGCTTCCGCAATATCTGGATTAAGTGTACCAACAACTCTTGGAACTTCTTCCGTACTGGCCCCGATGGAATCTACTTCATAGCCTCTGATGGTGGa                                                                                                                                                                                                                                          <  1:130137/301‑1 (MQ=255)`  
                                                                      `ggACTATGCAGTAGTTAACAAACACTTCCACGTAGGTCAGGCCGTTGTGGCCACTGATGGTAATATTCAAGGTACTAAGTGGGGAGGTAAATGGCTGGATGCTTACCTACGTG‑ACAGCTTCGTTGCGAAGTCCAAGGCGTGGACTCAGGTGTGGTCTGGTAGTGCTGGCGGTGGGGTAAGTGTGACTGTTTCACAGGATCTCCGCTTCCGCAATATCTGGATTAAGTGTACCAACAACTCTTGGAACTTCTTCCGTACTGGCCCCGATGGAATCTACTTCATAGctgtctcttatacacat                                                                                                                                                                                                                                       >  1:105934/1‑285 (MQ=255)`  
                                                                      `ggACTATGCAGTAGTTAACAAACACTTCCACGTAGGTCAGGCCGTTGTGGCCACTGATGGTAATATTCAAGGTACTAAGTGGGGAGGTAAATGGCTGGATGCTTACCTACGTG‑ACAGCTTCGTTGCGAAGTCCAAGGCGTGGACTCAGGTGTGGTCTGGTAGTGCTGGCGGTGGGGTAAGTGTGACTGTTTCACAGGATCTCCGCTTCCGCAATATCTGGATTAAGTGTACAGACAACTCTTGGAACTTCTTCCGTACTGGCCCCGATGGAATCTACTTCATAGCCTCTGATGGTGGATgg                                                                                                                                                                                                                                       >  1:479586/1‑301 (MQ=255)`  
                                                                                              `gttcgaggTAGGGCAGGCTGTTGTGGCCACTGATGGTAATATTCAGGGTACTAAGTGGTGAGGTAAATGGCTGGATGCTCACCTACGTG‑ACAGCTTCGTTGCGAAGTCCAAGGCGTGGACACAGGTGTGGTCTGGTAGTGATGGCGGTGGGTTAAGTGTGACTGTTTAACAGGATCTCCGATTACGCAATATCTGGATTAAGTGTGCCAACAACTCTTGGAACTTCTTCCGTACTGGCCCCGATGGAATCTACTTCATAGCCTCTGATGGGGGATGGTTACGATTCCAAATACACTCCAAc                                                                                                                                                                                                               <  1:653710/294‑1 (MQ=255)`  
                                                                                              `cTTCCACGTAGGTCAGGCCGTTGTGGCCACTGATGGTAATATTCATGGTACTAAGTGGGGAGGTAAATGGCTGGATGCTTACCTACGTG‑ACAGCTTCGTTGCGAAGTCCAAGGCGTGGACTCAGGTGTGTTCTGGTAGTGCTGGCGGTGGGGTAAGTGTGACTGTTTCACTGGATCTCCGCTTCCGCAATATCTGGATTAAGTGTACCAACAACTCTTGGAACTTCTTCCGTACTGGCCCCGATGGACTCTACTTCATAGCCTCTGAGGGTGGATTGTTACGATTCCAAATACACTCCAAc                                                                                                                                                                                                               >  1:660946/1‑301 (MQ=255)`  
                                                                                              `cTTCCACGTAGGTCAGGCCGTTGTGGCCAATGATGGTAATATTCAAGGTACTAAGTGGGGAGGTAGATGGCTGGATGCTTACCTACGGG‑ACAGCGTCGTTGCGAAGTCCAAGGCGTGGACTCAGGTGTGGTCTGGTAGTGCTGGCGGTTGGGGAAGTGTGACTGTTTCACAGGATCTCCGCTTCCGCAATATCTGGATTAAGTGTGCCAACAACTCTTGGGACTTCTTCCGTACTGGCCCCGATGGAATCTACTTCATAGCCTCTGATGGTGGATGGTTACGATTCCAAATACACTCCAAc                                                                                                                                                                                                               <  1:26561/301‑1 (MQ=255)`  
                                                                                                       `agcaagggcggtgggggcaattGATGTTAATA‑TCAAGGGACTAAGGGGGGAGGTAAAAGGCTGGATGGTTACCTTAGTGAACGGCTTGGTTGGGAGGTCCAGGGGGTGGATCAAGGTGTGG‑GTGGTAAGGCTGGGGGTGGGGTTAATGTGACTGTTTCACAAGATCTCCGCTTTCGCAATTTCTTGATTAAGGTGGCCCACAACTCTTGGAACTTTTTCCGTAATGGCCCCGATGGAATCTACTTCATAGCCTCTGATGGTGGATGGTTACGATTCCAAATACACTCCAACGGTCTCGGAt                                                                                                                                                                                                     <  1:139996/280‑1 (MQ=255)`  
                                                                                                                `cGTTGTGGCCACTGATGGTAATATTCAAGGTACTAAGTGGGGAGGTAAATGGCTGGATGCTTACCTACGTG‑ACAGCTTCGTTGCGAAGTCCAAGGCGTGGACTCAGGTGTGGTCTGGTAGTGCTGGCGGTGGGGTAAGTTTGACTGTTTCACAGGATCTCCGCTTCCGCAATATCTGGATTAAGTGTACCAACAACTCTTGGAACTTCTTCCGTACTGGCCCCGATGGAATCTACTTCATAGCCTCTGATGGTGGATGGTTACGATTCCAAATACACTCCAACGGTCTCGGAGTCAAGAat                                                                                                                                                                                             <  1:607902/301‑1 (MQ=255)`  
                                                                                                                     `tgGCCACTGATGGTAATATTCAAGGTACTAAGTGGGGAGGTAAATGGCTGGATGCTTACCTACGTG‑ACAGCTTCGTTGCGAAGTCCAAGGCGTGGACTCAGGTGTGGTCTGGTAGTGCTGGCGGTGGGGTAAGTGTGGCTGTTTCACAGGATCTCCGCTTCCGCAATATCTGGATTAAGTGTACCAACAACTCTTGGAACTTCTTCCGTACTGGCCCCGATGGAATCTACTTCATAGCCTCTGATGGTGGATGGTTACGATTCCAAATACACTCCAACGGTCTCGGATTCAAGAATATTGc                                                                                                                                                                                        >  1:293554/1‑301 (MQ=255)`  
                                                                                                                      `ggCCACTGATGGTAATATTCAAGGTACTAAGTGGGGAGGTAAATGGCTGGATGCTTACCTACGTG‑ACAGCTTCGTTGCGAAGTCCAAGGCGTGGACTCAGGTGTGGTCTGGTAGTGCTGGCGGTGGGGTAAGTGTGACTGTTTCACAGGATCTCCGCTTCCGCAATATCTGGATTAAGTGTACCAACAACTCTTGGAACTTCTTCCGTACTGGCCCCGATGGAATCTACTTCATAGCCTCTGATGGTGGATGGTTACGATTCCAAATACACTCCAACGGTCTCGGATTCAGGAATATTTCa                                                                                                                                                                                       >  1:393370/1‑301 (MQ=255)`  
                                                                                                                           `ctgctgGTAATATTCAAGGTACTAAGTGGGGAGGTAAATGGCTGGATGCTTACCTACGTG‑ACAGCTTCGTTGCGAAGTCCAAGGCGTGGACTCAGGTGTGGTCTGGTAGTGCTGGCGGTGGGGTAAGTGTGACTGTTTCACAGGATCTCCGCTTCCGCAATATCTGGATTAAGTGTACCAACAACTATTGGAACTTCTTCCGTACTGGCCCCGATGGAATCTACTTCATAGCCTCTGATGGTGGATGGTTACGATTCCAAATACACTCCAACGGTCTCGGATTCAAGAATATTGCAGACAg                                                                                                                                                                                  >  1:259256/1‑301 (MQ=255)`  
                                                                                                                               `tGGTAATATTCAAGGTACTAAGTGGGGAGGTAAATGGCTGGATGCTTACCTACGTG‑ACAGCTTCGTTGCGAAGTCCAAGGCGTGGACTCAGGTGTGGTCTGGTAGTGCTGGCGGTGGGGTAAGTGTGACTGTTTCACAGGATCTCCGCTTCCGCAATATCTGGATTAAGTGTGCCAACAACTCTTGGAACTTCTTCCGTACTGGCCCCGATGGAATCTACTTCATAGCCTCTGATGGTGGATGGTTACGATTCCAAATACACTCCAACGGTCTCGGATTCAAGAATATTGCAGACagtagt                                                                                                                                                                              >  1:512686/1‑301 (MQ=255)`  
                                                                                                                                 `gTAATATTCAAGGTACTAAGTGGGGAGGTAAATGGCTGGATGCTTACCTACGTG‑ACAGCTTCGTTGCGAAGTCCAAGGCGTGGACTCAGGTGTGGTCTGGTAGTGCTGGCGGTGGGGTAAGTGTGACTGTTTCACAGGATCTCCGCTTCCGCAATATCTGGATTAAGTGTACCAACAACTCTTGGAACTTCTTCCGTACTGGCCCCGATGGAATCTACTTCATAGCCTCTGATGGTGGATGGTTACGATTCCAAATACACTCCAACGGTCTCGGATTCAAGAATATTGCAGACATTCGTTc                                                                                                                                                                            >  1:486550/1‑301 (MQ=255)`  
                                                                                                                                   `aaTATTCAAGGTAATAAGTGTGGAGGTAAATGGCTGGATGCTTACCTACGTG‑ACAGCTTCGTTGCGAAGTCCAAGGCGTGGACTCAGGTGTGGTCTGGTAGTGCTGGCGGTGGGGTAAGTGTGACTGTTTCACAGGATCTCCGCTTCCGCAATATCTGGATTAAGTGTGCCAACAACTCTTGGAACTTCTTCCGTACTGGCCCCGATGGAATCTACTTCATAGCCTCTGATGGTGGATGGTTACGATTCCAAATACACTCCAACGGTCTCGGATTCAAGAATATTGCAGACAGTCGTTCAg                                                                                                                                                                          <  1:158262/301‑1 (MQ=255)`  
                                                                                                                                    `atatTCAAGGTACTAAGTGGGGAGGTAAATGGCTGGATGCTTACCTACGTG‑ACAGCTTCGTTGCGAAGTCCAAGGCGTGGACTCAGGTGTGGTCTGGTAGTGCTGGCGGTGGGGTAAGTGTGACTGTTTCACAGGATCTCCGCTTCCGCAATATCTGGATTAAGTGTGCCAACAACTCTTGGAACTTCTTCCGTACTGGCCCCGATGGAATCTACTTCATAGCCTCTGATGTTGGATGGTTACGATTCCAAATACACTCCAACGGTCTCGGATTCAAGAATATTGCAGACAGTCGTTCAGt                                                                                                                                                                         >  1:52803/1‑301 (MQ=255)`  
                                                                                                                                     `tatTCAAGGTACTAAGTGGGGAGGTAAATGGCTGGATGCTTACCTACGTG‑ACAGCTTCGTTGCGAAGTCCAAGGCGTGGACTCAGGTGTGGTCTGGTAGTGCTGGCGGTGGGGTAAGTGTGACTGTTTCACAGGATCTCCGCTTCCGCAATATCTGGATTAAGTGTGCCAACAACTCTTGGAACTTCTTCCGTACTGGCCCCGATGGAATCTACTTCATAGCCTCTGATGGTGGATGGTTACGATTCCAAATACACTCCAACGGTCTCGGATTCAAGAATATTGCAGACAGTCGTTCAGTa                                                                                                                                                                        >  1:601115/1‑301 (MQ=255)`  
                                                                                                                                     `tatTCAAGGTACTAAGTGGGGAGGTAAATGGCTGGATGCTTACCTACGTG‑ACAGCTTCGTTGCGAAGTCCAAGGCGTGGACTCAGGTGTGGTCTGGTAGTGCTGGCGGTGGGGTAAGTGTGACTGTTTCACAGGATCTCCGCTTCCGCAATATCTGGATTAAGTGTACCAACAACTCTTGGAACTTCTTCCGTACTGGCCCCGATGGAATCTACTTCATAGCCTCTGATGGTGGAAGGTTACGATTCCAAATACACTCCAACGGTCTCGGATTCAAGAATATTGCAGACAGTCGTTCAGTa                                                                                                                                                                        >  1:613562/1‑301 (MQ=255)`  
                                                                                                                                      `atTCAAGGTACTAAGTGGGGAGGTAAATGGCTGGATGCTTACCTACGTG‑ACAGCTTCGTTGCGAAGTCCAAGGCGTGGACTCAGGTGTGGTCTGGTAGTGCTGGCGGTGGGGTAAGTGTGACTGTTTCACAGGATCTCCGCTTCCGCAATATCTGGATTAAGTGTGCCAACACCTCTTGGAACTTCTTCCGTACTGTCCCCGATGGAATCTACTTCATAGCCTCTGATGGTGGATGGTTACGATTCCAAATACACTCAAACGGTCTCGGATTCAAGAATATTGCAGATAGTCGTTCAGTAc                                                                                                                                                                       >  1:234673/1‑301 (MQ=255)`  
                                                                                                                                      `agacatggTACTAAGTGGGGAGGTAAATGGCTGGATGATTACCTACGTG‑ACAGCTTCGTTGCGAAGGCCAAGTCGTGGACTCAGGTGTGGTCTGGTAGTGCTGGCGGTGGGGTAAGTGTGACTGTTTCACAGGATCTCCGCGTCCGCAATATCTGGATTAAGTGTGCCAACAACTCTTGGAACTTCTTCCGTACTGGCCCCGATGGAATCTACTTCATAGCCTCTGATGGTGGATGGTTACGATTCCAAATACACTCCAACGGTCTCGGATTCAAGAATATTGCAGACAGTCGTTCAGTAc                                                                                                                                                                       <  1:400597/295‑1 (MQ=255)`  
                                                                                                                                         `cAAGGTACTAAGTGGGGAGGTAAATGGCTGGATGATTACCTACGTG‑ACAGCTTCGTTGCGAAGTCCAAGGCGTGGACTCAGGTGTGGTCTGGTAGTGCTGGCGGTGGGGTAAGTGTGACTGGTTCACAGGATCTCCGCTTCCGCAATATCTGGATTAAGTGTATCAACAACTCTTGGAACTTCTTCCGTACTGGCCCCGATGGAATCTACTTCATAGCCTCTGATGGTGGATGGTTACGATTCCAAATACACTCCAACGGTCTCGGATTCAAGAATATTGCAGACAGTCGTTCAGTACCTa                                                                                                                                                                    <  1:287863/301‑1 (MQ=255)`  
                                                                                                                                           `atgTACTAAGTGGGGAGGTAAATGGCTGGATGCTTACCTACGTG‑ACAGATTAGTTGCGAAGTCCAAGGCGTGGACTCAGGTGTGGTATGGTAGTGCTGTCGGTGGGGTAAGTGTGACTGTTTCACAGGATCTCCGCTTCCGCAATATCTGGATTAAGTGTACCAACAACTCTTGGAACTTCTTCCGTACTGGCCCCGATGGAATCTACTTCATAGCCTCTGATGGTGGATGGTTACGATTCCAAATACACTCCAACGGTCTCGGATTCAAGAATATTGCAGACAGTCGTTCAGTACCTAAt                                                                                                                                                                  <  1:318385/299‑1 (MQ=255)`  
                                                                                                                                           `aGGTACTAAGTGGGGAGGTAAAGGGCTGGATGCTTACCTACGTG‑ACAGCTGCGTTGCGAAGTCCAAGGCGTGGACTCAGGTGTGGTCTGGTAGTGCTGGCGGTGGTGTAAGTGTGACTGTTTCACAGGATCTCCGCTTCCGCAATATCTGGATTAAGTGTACCAACAACTCTTGGAACTTCTTCCGTACTGGCCCCGATGGAATCTACTTCATAGCCTCTGATGGTGGATGGTTACGATTCCAAATACACTCCAACGGTCTCGGATTCAAGAATATTGCAGACAGTCGTTCAGTACCTAAt                                                                                                                                                                  <  1:439358/301‑1 (MQ=255)`  
                                                                                                                                            `ggTACTAAGTGGGGAGGTAAATGGCTGGATGCTTACCTACGTG‑ACAGCTTCGATGCGAAGTCCAAGGCGTGGACTCAGGTGTGGTCTGGTAGTACTGGCGGTGGGGTAAGTGTGACTGTATCACAGGATCGCCGCTTCCGTAATATCTGGATTAAGTGTGACAACAACTCTTGGGACTACTTCCGTACTGCACCCGATGGCATCTACTTCATACCCTCTGTTGGTGGAGGGTTACGATTCACAATAAACTCCAACGGTCTCAGATTCAAGAACATTGCAGACAGTCGTTCTGTACATAATg                                                                                                                                                                 >  1:163858/1‑301 (MQ=255)`  
                                                                                                                                             `gTACTAAGTGGGGAGGTAAATGGCTGGATGCGTACCTACGTG‑ACAGCTTCGTTGCGAAGTCCAAGGCGTGGACTCATGTGTGGTCTGGTAGTGCTGGCGGTGGGGTAAGTGTGACTGTTTCACAGGATCTCCGCTTCCGCAATATCTGGATTAAGTGTACCAACAACTCTTGGAACTTCTTCCGTACTGGCCCCGATGGAATCTACTTCATAGCCTCTGATGGTGGATGGTTACGATTCCAAATACACTCCAACGGTCTCGGATTCAAGAATATTGCAGACAGTCGTTCAGTACCTAATGc                                                                                                                                                                <  1:168290/301‑1 (MQ=255)`  
                                                                                                                                                 `tAAGTGGGGAGGTAAATGGCTGGATGCTTACCTACGTG‑ACAGCTTCGTTGCGAAGTCCAAGGCGTGGACTCAGGTGTGGTCTGGTAGTGCTGGCGGTGGGGTAAGTGTGACTGTTTCACAGGATCTCCGCTTCCGCACTATCTGGATTAAGTGTGCCAACAACTCTTGGAACTTCTTCCGTACTGTCCCCGATAGAATCTACTTCATAGCCTCTGATGGTGGATTGTTACGATTCCAAATACACTCCAACGGTCTCGGATTCAAGAATATTGCAGACAGTCGTTCAGTACCTAATGCAATc                                                                                                                                                            >  1:58047/1‑301 (MQ=255)`  
                                                                                                                                                  `gggacaggAGGTAAATGGCTGGATGCTTACCTACGTG‑ACAGCTTCGTTGCGAAGTCCAAGGCGTGGACTCAGGTGTGGTCTGGTAGTGCTGGCGGTGGGGTAAGTGTGACTGTTTCACAGGATCTCCGCTTCCGCAATATCTGGATTAAGTGTACCAACAACTCTTGGAACTTCTTCCGTACTGGCCCCGATGGAATCTACTTCATAGCCTCTGATGGTGGATGGTTACGATTCCAAATACACTCCAACGGTCTCGGATTCAAGAATATTGCAGACAGTCGTTCAGTACCTAATGCAATCa                                                                                                                                                           <  1:241351/295‑1 (MQ=255)`  
                                                                                                                                                     `gggggAGGTAAATGGCTGGATGCTTACCTACGGG‑ACAGCTTCGTTGCGAAGTCCAAGGCGTGGACTCAGGTGTGGTCTGGTAGTGCTGGCGGTGGGGTAAGTGTGACTGTTTCACAGGATCTCCGCTTCCGCAATATCTGGATTAAGTGTACCAACAACTCTTGGAACTTCTTCCGTACTGGCCCCGATGGAATCTACTTCATAGCCTCTGATGGTGGATGGTTACGATTCCAAATACACTCCAACGGTCTCGGATTCAAGAATATTGCAGACAGTCGTTCAGTACCTAATGCAATCAtgg                                                                                                                                                        <  1:419439/300‑1 (MQ=255)`  
                                                                                                                                                         `gAGGTAAATGGCTGGATGCTTACCTACGTG‑ACAGCTTCGTTGCGAAGTCCAAGGCGTGGACTCAGGTGTGGTCTGGTAGTGCTGGCGGTGGGGTAAGTGTGACTGTTTCACAGGATCTCCGCTTCCGCAATATCTGGATTAAGTGTACCAACAACTCTTGGAACTTCTTCCGTACTGGCCCCGATGGAATCTACTTCATAGCCCCTGATGGTGGATGGTTACGATTCCAAATACACTCCAACGGTCTCGGATTCAAGAATATTGCAGACAGTCGTTCAGTACCTAATGCAATCATGGTGga                                                                                                                                                    >  1:193457/1‑301 (MQ=255)`  
                                                                                                                                                           `ggTAAATGGCTGGATGCTTACCTACGTG‑ACAGCTTCGTTGCGAAGTCCAAGGCGTGGACTCAGGTGTGGTCTGGTAGTGCTGGCGGTGGGGTAAGTGTGACTGTTTCACAGGATCTCCGCTTCCGCAATATCTGGATTAAGTGTGCCAACAACTCTTGGAACTTCTTCCGTACTGGCCCCGATGGAATCTACTTCATAGCCTCTGATGGTGGATGGTTACGATTCCAAATACACTCCAACGGTCTCGGATTCAAGAATATTGCAGCCAGTCGTTCAGTACCTAATGCAATCATGGTGgaga                                                                                                                                                  >  1:561659/1‑301 (MQ=255)`  
                                                                                                                                                                        `aTGCTTACCTACGTG‑ACAGCTTCGTTGCGAAGTCCAAGGCGTGGACTCAGGTGTGGTCTGGTAGTGCTGGCGGTGGGGTAAGTGTGACTGTTTCACAGGATCTCCGCTTCCGCAATATCTGGATTAAGTGTGCCAACAACTCTTGGAACTTCTTCCGTACTGGCCCCGATGGAACCTACTTCATCGCCTCTGATGGTGGATGGTTACGATTCCAAATCCACTCCAACGGTCTCGGATTCAAGAATATTGCAGACAGTCGTTCAGTACCTAATGCAATCAtggtggctgtctcttatacacc                                                                                                                                     >  1:425861/1‑285 (MQ=255)`  
                                                                                                                                                                               `ccTACGTG‑ACAGGTTCGTTGCGAAGTCCAAGGCGTGGACTCAGGTGTTGTCTGGTAGGGCTGGCGGTGGGGTAAGTGTGACTGGTTCACAGGATCTCCGCTTCCGCAATATCTGGATTAAGTGTACCAACAACTCTTGGAACTTCTTCCGTACTGGCCCCGATGGAATCTACTTCATAGCCTCTGATGGTGGATGGTTACGATTCCAAATACACTCCAATGGTCTCGGATTCAAGAATATTGCAGACAGTCGTTCAGTACCTAATGCAATCATGGTGGAGAACGAGTAATTGGTAAATcac                                                                                                                              <  1:61991/301‑1 (MQ=255)`  
                                                                                                                                                                                  `aCGTG‑ACAGCTTCGTTGCGAAGTCCAAGGCGTGGACTCAGGTGTGGTCTGGTAGTGCTGGCGGTGGGGTAAGTGTGACTGTTTCACAGGATCTCCGCTTCCGCAATATCTGGATTAAGTGTGCCAACAACTCTTGGAACTTCTTCCGTACTGGCCCCGATGGAATCTACTTCATAGCCTCTGATGGTGGATGGTTACGATTCCAAATACACTCCAACGGTCTCGGATTCAAGAATATTGCAGACAGTCGTTCAGCACCTAATGCAATCATGGTGGAGAACGAGTAATTGGTAAATCACAAg                                                                                                                           >  1:205128/1‑301 (MQ=255)`  
                                                                                                                                                                                  `aCGTG‑ACAGCTTCGTTGCGAAGTCCAAGGCGTGGACTCAGGTGTGGTCTGGTAGTGCTGGCGGTGGGGTAAGTGTGACTGTTTCACAGGATCTCCGCTTCCGCAATATCTGGATTAAGTGTACCAACAACTCTTGGAACTTCTTCCGTACTGGCTCCGATGGAATCTACTTCATAGCCTCTGATGGTGGATGGTTACGATTCCAAATACACTCCAACGGTCTCGGATTCAAGAATATTGCAGACAGTCGTTCAGTACCTAATGCAATCATGGTGGAGAACGAGTAATGGGTAAATCACAAg                                                                                                                           >  1:363428/1‑301 (MQ=255)`  
                                                                                                                                                                                   `cGTG‑ACAGCTTCGTTGCGAAGTCCAAGGCGTGGAATCAGGTGTGGTCTGGTAGTGCTGGCGGTGGGGTAAGTGTGACTGTTTCACAGGATCTCCGCTTCCGCAATATCTGGATTAAGTGTGCCAACAACTCTTGGAACTTCTTCCGTACTGGCCCCGATGGAATCTACTTCATAGCCTCTGATGGTGGATTGTTACGATTCCAAATACACTCCAACGGTCTCGGATTCAAGAATATTGCAGACAGTCGTTCAGTACCTAATGCAATCATGGTGGAGAACGAGTAATTGGTAAATCACAAgg                                                                                                                          >  1:499122/1‑301 (MQ=255)`  
                                                                                                                                                                                           `gCTTCGTTGCGAAGTCCAAGGCGTGGACTCAGGTGTGGTCTGGTAGTGCTGGCGGTGGGGTAAGTGTGACTGTTTCACAGGATCTCCGCTTCCGCAATATCTGGATTAAGTGTGCCAACAACTCTTGGAACTTCTTCCGTACTGGCCCCAATGGAATCTACTTCATAGCCTCTGATGGTGGATGGTTACGATTCCAAATACACTCCAACGGTCTCGGATTCAAGAATATTGCAGACAGTCGTTCAGTACCTAATGCAATCATGGTGGAGAACGAGTAATTGGTAAATCACAAGGAAAGACg                                                                                                                   <  1:9341/301‑1 (MQ=255)`  
                                                                                                                                                                                                  `tGCGAAGTCCAAGGCGTGGACTCAGGTGTGGTCTGGTAGTGCTGGCGGTGGGGTAAGTGTGACTGTTTCACAGGATCTCCGCTTCCGCAATATCTGGATTAAGTGTACCAACAACTCTTGGAACTTCTTCCGTACTGGCCCCGATGGAATCTAATTCATAGCCTCTGATGGTGGATGGTTACGATTCCAAATACACTCCAACGGTCTCGGATTCAAGAATATTGCAGACAGTCGTTCAGTACCTAATGCAATCATGGTGGAGAACGAGTAATTGGTAAATCACAAGGAAAGACGTGTAGTc                                                                                                            <  1:277042/301‑1 (MQ=255)`  
                                                                                                                                                                                                        `gTCCAAGGCGTGGACTCAGGTGTGGTCTGGTAGTGCTGGCGGTGGGGTAAGTGTGACTGTTTCACAGGATCTCCGCTTCCGCAATATCTGGATTAAGTGTACCAACAACTCTTGGAACTTCTTCCGTACTGGCCCCGATGGAATCTACTTCATAGCCTCTGATGGTGGATGGTTACGATTCCAAATACACTCCAACGGTCTCGGATTCAAGAATATTGCAGACAGTCGTTCAGTACCTAATGCAATCATGGTGGAGAACGAGTAATTGGTAAATCACAAGGAAAGACGTGTAGTcctgtct                                                                                                      >  1:647018/1‑296 (MQ=255)`  
                                                                                                                                                                                                                   `ggACTCAGGTGTGGTCTGGTAGTGCTGGCGGTGGGGTAAGTGTGACTGTTTCACAGGATCTCCGCTTCCGCAATATCTGGATTAAGTGTACCAACAACTCTTGGAACTTCTTCCGTACTGGCCCCGATGGAATCTACTTCATAGCCTCTGATGGTGGATGGTTACGATTCCAAATACACTCCAACGGTCTCGGATTCAAGAATATTGCAGACAGTCGTTCAGTACCTAATGCAATCATGGTGGAGAACGAGTAATTGGTAAATCACAAGGAAAGACGTGTAGTCCACGGATGGActcctgt                                                                                           >  1:567658/1‑297 (MQ=255)`  
                                                                                                                                                                                                                     `gacagggTGTGGTCTGGTAGTGCTGGCGGTGGGGTAAGTGTGACTGTTTCACAGGATCTCCGCTTCCGCAATATCTGGATTAAGTGTACCAACAACTCTTGGAACTTCTTCCGTACTGGCCCCGATGGAATCTACTTCATAGCCTCTGATGGTGGATGGTTACGATTCCAAATACACTCCAACGGTCTCGGATTCAAGAATATTGCAGACAGTCGTTCAGTACCTAATGCAATCATGGTGGAGAACGAGTAATTGGTAAATCACAAGGAAAGACGTGTAGTCCACGGATGGACTCTCAagg                                                                                         <  1:387197/296‑1 (MQ=255)`  
                                                                                                                                                                                                                     `aCTCAGGTGTGGTCTGGTAGTGCTGGCGGTGGGGTAAGTGTGACTGTTTCACAGGATCTCCGCTTCCGCAATATCTGGATTAAGTGTGCCAACAACTCTTGGAACTTCTTCCGTACTGGCCCCGATGGAATCTACTTCATAGCCTCTGATGGTGGATGGTTACGATTCCAAATACACTCCAACGGTCTCGGATTCAAGAATATTGCAGACAGTCGTTCAGTACCTAATGCAATCATGGTGGAGAACGAGTAATTGGTAAATCACAAGGAAAGACGTGTAGTCCACGGATGGACTCTCAagg                                                                                         <  1:517747/301‑1 (MQ=255)`  
                                                                                                                                                                                                                      `cTCAGGTGTGGTCTGGTAGTGCTGGCGGTGGGGTAAGTGTGACTGTTTCACAGGATCTCCGCTTCCGCAATATCTGGATTAAGTGTACCAACAACTCTTGGAACTTCTTCCGTACTGGCCCCGATGGAATCTACTTCATAGCCTCTGCTGGTGGATGGTTACGATTCCAAATACACTCCAACGGTCTCGGATTCAAGAATATTGCAGACAGTCGTTCAGTACCTAATGCAATCATGGTGGAGAACGAGTAATTGGTAAATCACAAGGAAAAACGTGTAGTCCACGGATGGACTCTCAaggg                                                                                        >  1:228197/1‑300 (MQ=255)`  
                                                                                                                                                                                                                      `cTCAGGTGTGGTCTGGTAGTGCTGGCGGTGGGGTAAGTGTGACTGTTTCACAGGATCTCCGCTTCCGCAATATCTGGATTAAGTGTACCAACAACTCTTGGAACTTCTTCCGTACTGGCCCCGATGGAATCTACTTCATAGCCTCTGATGGTGGATGGTTACGATTCCAAATACACTCCAACGGTCTCGGATTCAAGAATATTGCAGACAGTCGTTCAGTACCTAATGCAATCATGGTGGAGAACGAGTAATTGGTAAATCACAAGGAAAGACGTGTAGTCCACGGATGGACTCTCAagga                                                                                        <  1:458117/301‑1 (MQ=255)`  
                                                                                                                                                                                                                         `gggattgttttGGTAGGGCTGGCGTTGGGGTATGTTTGACTGTTTCACAGGATCTCCGCTTCCGCAATATCTGGATTAAGTGTACCAACAACTCTTGGAACTTCTTCCGTACTGGCCCCGATGGAATCTACTTCATAGCCTCTGATGGTGGATGGTTACGATTCCAAATACACTCCAACGGTCTCGGATTCAAGAATATTGCAGACAGTCGTTCAGTACCTAATGCAATCATGGTGGAGAACGAGTAATTGGTAAATCACAAGGAAAGACGTGTAGTCCACGGATGGACTCTCAAGGAGGt                                                                                     <  1:125526/291‑1 (MQ=255)`  
                                                                                                                                                                                                                         `aGGTGTGGTCTGGTATTGCTGGCGGTGGGGTAAGTGTGACTGTTTCACAGGATCTCCGCTTCCGCAATATCTGGATTAAGTGTACCAACAACTCTTGGAACTTCTTCCGTACTGGCCCCGATGGAATCTACTTCATAGCCTCTGATGGTGGATGGTTACGATTTCAAATACACTCCAACGGTCTCGGATTCAAGAATATTGCAGACAGTCGTTCAGTACCTAATGCAATCATGGTGGAGAACGAGTAATTGGTAAATCACAAGGAAAGACGTGTAGTCCACGGATGGACTCTCAAGGAGGt                                                                                     <  1:282299/301‑1 (MQ=255)`  
                                                                                                                                                                                                                         `aGGTGTGGTCTGGTAGTGCTGGCGGTGGGGTAAGTGTGACTGTTTCACAGGATCTCCGCTTCCGCAATATCTGGATTAAGTGTACCAACAACTCTTGGAACTTCTTCCGTACTGGCCCCGATGGAATCTACTTCATAGCCTCTGATGGTGGATGGTTACGATTCCAAATACACTCCAACGGTCTCGGATTCAAGAATATTGCAGACAGTCGTTCAGTACCTAATGCAATCATGGTGGAGAACGAGTAATTGGTAAATCACAAGGAAAGACGTGTAGTCCACGGATGGACTCTCAAGGAGGt                                                                                     <  1:667884/301‑1 (MQ=255)`  
                                                                                                                                                                                                                          `ggTGTGGTCTGGTAGTGCTGGCGGTGGGGTAAGTGTGACTGTTTCACAGGATCTCCGCTTCCGCAATATCTGGATTAAGTGTGCCAACAACTCTTGGAACTTCTTCCGTACTGGCCCCGATGGAATCTACTTCATAGCCTCTGATGGTGGATGGTTACGATTCCAAATACACTCCAACGGTCTCGGATTCAAGAATATTGCAGACAGTCGTTCAGTACCTAATGCAATCATGGTGGAGAACGAGTAATTGGTAAATCACAAGGAAAGACGTGTAGTCCACGGATGGACTCTCAAGGAGGTa                                                                                    >  1:11244/1‑301 (MQ=255)`  
                                                                                                                                                                                                                           `gtgtGGTCTGGTAGTGCTGGCGGTGGGGTAAGTGTGACTGTTTCACAGGATCTCCGCTTCCGCAATATCTGGATTAAGTGTGCCAACAACTCTTGGAACTTCTTCCGTACTGGCCCCGATGGAATCTACTTCATAGCCTCTGATGGTGGATGGTTACGATTCCAAATACACTCCAACGGTCTCGGATTCAAGAATATTGCAGACAGTCGTTCAGTACCTAATGCAATCATGGTGGAGAACGAGTAATTGGTAAATCACAAGGAAAGACGTGTAGTCCACGGATGGACTCTCAAGGAGGTAc                                                                                   <  1:493131/301‑1 (MQ=255)`  
                                                                                                                                                                                                                               `tgTCTGCTGGTGCTGTCGGTGTCGTAAGTCTTACTGTTTCACAGTATCTCCGCTTCCGCACTATCTGGATTAAGTGTGCCACCAACTCTTGGTACGTCTTCCGTTCTGGCCCCGACTGAATCTACTTCATAGCCTCTGATGGTGTATGGTTACGATTCCAAATACACTCCAACGTTCTCGGATTCAAGAATATTGCAGACAGTCGTTCAGTACCTAATGCAATCATGGTGGAGAACGAGTAATTGGTAAATCACAAGGAAAGACGTTTAGTCCACGGATGGACTCTCAAGGAGGTACAAgt                                                                               <  1:2098/300‑2 (MQ=255)`  
                                                                                                                                                                                                                                `gTCTGGTAGTGCTGGCGGTGGGGTAAGTGTGACTGTTTCACAGGATCTCCGCTTCCGCAATATCTGGATTAAGTGTGCCAACAACTCTTGGAACTTCTTCCGTACTGGCCCCGATGGAATCTACTTCATAGCCTCTGATGGTGGATGGTTACGATTCCAAATACACTCCAACGGTCTCGGATTCAAGAATATTGCAGACAGTCGTTCAGTACCTAATGCAATCATGGTGGAGAACGAGTAATTGGTAAATCACAAGGAAAGACGTGTAGTCCACGGATGGACTCTCAAGGAGGTACAAGGt                                                                              >  1:594054/1‑301 (MQ=255)`  
                                                                                                                                                                                                                                `gTCTGGTAGTGCTGGCGGTGGGGTAAGTGTGACTGTTTCACAGGATCTCCGCTTCCGCAATATCTGGATTAAGTGTACCAACAACTCTTGGAACTTCTTCCGTACTGGCCCCGATGGAATCTACTTCATAGCCTCTGATGGTGGATGGTTACGATTCCAAATACACTCCAACGGTCTCGGATTCAAGAATATTGCAGACAGTTGTTCAGTACCTAATGCAATCATGGTGGAGAACGAGTAATTGGTAAATCACAAGGAAAGACGTGTAGTCCACGGATGGACTCTCAAGGAGGTACATGGt                                                                              >  1:188439/1‑301 (MQ=255)`  
                                                                                                                                                                                                                                `gTCTGGTAGTGCTGGCGGTGGGGTAAGTGTGACTGTTTCACAGGATCTCCGCTTCCGCAATATCTGGATTAAGTGTACCAACAACTCTTGGAACTTCTTCCGTACTGGCCCCGATGGAATCTACTTCATAGCCTCTGATGGTGGATGGTTACGATTCCAAATACACTCCAACGGTCTCGGATTCAAGAATATTGCAGACAGTCGTTCAGTACCTAATGCAATCATGGTGGAGAACGAGTAATTGGTAAATCACAAGGAAAGACGTGTAGTCCACGGATGGACTCTCAAGGAGGTACAAGGt                                                                              <  1:586870/301‑1 (MQ=255)`  
                                                                                                                                                                                                                                `gTCTGGTAGTGCTGGCGGTGGGGTAAGTGTGACTGTTTAACAGGATCTCCGCTTCCGCAATATCTGGATTAAGTGTACCAACAACTCTTGGAACTTCTTCCGTACTGGCCCCGATGGAATCTACTTCATAGCCTCTGATGGTGGATGGTTACGATTCCAAATACACTCCAACGGTCTCGGATTCAAGAATATTGCAGACAGTCGTTCAGTACCTAATGCAATCATGGTGGAGAACGAGTAATTGGTAAATCACAAGGAAAGACGTGTAGTCCACGGATGGACTCTCAAGGAGGTACAAGGt                                                                              <  1:254145/301‑1 (MQ=255)`  
                                                                                                                                                                                                                                  `cTGGTAGTGCTGGCGGTGGGGTAAGTGTGACTGTTTCACAGGATCTCCGCTTCCGCAATATCTGGATTAAGTGTGCCAACAACTCTTGGAACTTCTTCCGTACTGGCCCCGATGGAATCTACTTCATAGCCTCTGATGGTGGATGGTTACGATTCCAAATACACTCCAACGGTCTCGGATTCAAGAATATTGCAGACAGTCGTTCAGTACCTAATGCAATCATGGTGGAGAACGAGTAATTGGTAAATCACAAGGAAAGACGTGTAGTCCACGGATGGACTCTCAAGGAGGTACaactgtc                                                                            >  1:432249/1‑296 (MQ=255)`  
                                                                                                                                                                                                                                   `tGGTATTGCTGGCGGTGGGGTAAGTGTGACTGTTTCACAGGATCTCCGCTTCCGCAATATCTGGATTAAGTGTGCCAACAACTCTTGGAACTTCTTCCGTACTGGCCCCGATGGAATCTACTTCATAGCCTCTGATGGTGGATGGTTACGATTCCAAATACACTCCAACGGTCTCGGATTCAAGAATATTGCAGACAGCCGTTCAGTACCTAATGCAATCATGGTGGAGAACGAGTAATTGGTATATCACAAGGAAAGACGTATAGTCCACGGAGGGACTCTAAAGGAGGTACAAggatgt                                                                           >  1:374110/1‑297 (MQ=255)`  
                                                                                                                                                                                                                                    `ggTAGTGCTGGCGGTGGGGTAAGTGTGACTGTTTCACAGGATCTCCGCTTCCGCAATATCTGGATTAAGTGTACCAACAACTCTTGGAACTTCTTCCGTACTGGCCCCGATGGAATCTACTTCATAGCCTCTGATGGTGGATGGTTACGATTCCAAATACACTCCAACGGTCTCGGATTCAAGAATATTGCAGACAGTCGTTCAGTACCTAATGCAATCATGGTGGAGAACGAGTAATTGGTAAATCACAAGGAAAGACGTTTAGTCCACGGATGGACTCGCAAGGAGGTACAAGGTGCTa                                                                          >  1:522682/1‑301 (MQ=255)`  
                                                                                                                                                                                                                                             `ggCGGTGGGGTAAGTGTGACTGTTTCACAGGATCTCCGCTTCCGCAATATCTGGATTAAGTGTACCAACAACTCTTGGAACTTCTTCCGTACTGGCCCCGATGGAATCTACTTCATAGCCTCTGATGGTGGATGGTTACGATTCCAAATACACTCCAACGGTCTCGGATTCAAGAATATTGCAGACAGTCGTTCAGTACCTAATGCAATCATGGTGGAGAACGAGTAATTGGTAAATCACAAGGAAAGACGTGTAGTCCACGGATGGACTCTCAAGGAGGTACAAGGTGCTATCATTAGAc                                                                 <  1:8193/301‑1 (MQ=255)`  
                                                                                                                                                                                                                                              `gCGGTGTGGTAAGTGTGACTGTTTCACAGGATCTACGCTTCCGCAATATCTGGATTAAGTGTGCCAACAACTCTTGGAACTTCTTCCGTACTGGCCCCGATGGAATCTACTTCATAGCCTCTGATGGTGGATGGTTACGATTCCAAATACACTCCAACGGTCTCGGATTAAAGAATATTGCAGACAGTCGTTCAGTACCTAATGCAATCATGGTGGAGAACGAGTAATTGGTAAATCACAAGGAAAGACGTGTAGTCCACGGATGGACTCTCAAGGAGGTACAAGGTGCTATCATTAGACt                                                                <  1:368001/301‑1 (MQ=255)`  
                                                                                                                                                                                                                                                   `ggggTAAGTGTGACTGTTTCACAGGATCTCCGCTTCCGCAATATCTGGATTAAGTGTACCAACAACTCTTGGAACTTCTTCCGTACTGGCCCCGATGGAATCTACTTCATAGCCTCTGATGGTGGATGGTTACGATTCCAAATACACTCCAACGGTCTCGGATTCAAGAATATTGCAGACAGTCGTTCAGTACCTAATGCAATCATGGTGGAGAACGAGTAATTGGTAAATCACAAGGAAAGACGTGTAGTCCACGGATGGACTCTCAAGGAGGTACAAGGTGCTATCATTAGACTTTaac                                                           <  1:29955/301‑1 (MQ=255)`  
                                                                                                                                                                                                                                                   `ggggTAAGTGTGACTGTTTCACAGGATCTCCGCTTCCGCAATATCTGGATTAAGTGCGCCAACAACTCTTGGAACTTCTTCCGTACTGGCCCCGATGGAATCTACTTCATAGCCTCTGATGGTGGATGGTTACGATTCCAAATACACTCCAACGGTCTCGGATTCAAGAATATTGCAGACAGTCGTTCAGTACCTAATGCAATCATGGTGGAGAACGAGTAATTGGTAAATCACAAGGAAAGACGTGTAGTCCACGGATGGACTCTCAAGGAGGTACAAGGTGCTATCATTAGACTTTaac                                                           >  1:100069/1‑301 (MQ=255)`  
                                                                                                                                                                                                                                                           `tgtgACTGTTTCACAGGATCTCCGCTTCCGCAATATCTGGATTAAGTGTGCCAACAACTCTTGGAACTTCTTCCGTACTGGCCCCAATGGAATCTACTTCATAGCCTCTGATGGTGGATGGTTACGATTCCAAATACACTCCAACGGTCTCGGATTCAAGAATATTGCAGACAGTCGTTCAGTACCTAATGCAATCATGGTGGAGAACGAGTAATTGGTAAATCACAAGGAAAGACGTGTAGTCCACGGATGGACTCTCAAGGAGGTACAAGGTGCTATCATTaaagtctaatgatagcac                                                   <  1:406730/301‑18 (MQ=255)`  
                                                                                                                                                                                                                                                                  `gTTTCACAGGATCTCCGCTTCCGCAATATCTGGATTAAGTGTGCCAACAACTCTTGGAACTTCTTCCGTACTGGCCCCGATGGAATCTACTTCATAGCCTCTGATGGTGGATGGTTACGATTCCAAATACACTCCAACGGTCTCGGATTCAAGAATATTGCAGACAGTCGTTCAGTACCTAATGCAATCATGGTGGAGAACGAGTAATTGGTAAGTCACAAGGAAAGACGTGTAGTCCACGGATGGACTCTCAAGGAGGTACAAGGTGCTATCATTAGACTTTAACAACGAATTGATTAAg                                            >  1:345946/1‑301 (MQ=255)`  
                                                                                                                                                                                                                                                                  `gTTTCACAGGATCTCCGCTTCCGCAATATCTGGATTAAGTGTACCAACAACTCTTGGAACTTCTTCCGTACTGGCCCCGATGGAATCTACTTCATAGCCTCTGCTGGTGGATGGTTACGATTCCAAATACACTCCAACGGTCTCGGATTCAAGAATATTGCAGACAGTCGTTCAGTACCTAATGCAATCTTGGTGGAGAACGAGTAATTGGTAAATCACAAGGAAAGACGTTTAGTCCACGGATGGACTCTCGAGGAGGTACAAGGTGCTATCATTAGACTTTAACAACGAATTGATTAAg                                            >  1:357618/1‑301 (MQ=255)`  
                                                                                                                                                                                                                                                                  `gTTTCACAGGATCTCCGCTTCCGCAATATCTGGATTAAGTGTACCAACAACTCTTGGAACTTCTTCCGTACTGGCCCCGATGGAATCTACTTCATAGCCTCTGATGGTGGATGGTTACGATTCCAAATACACTCCAACGGTCTCGGATTAAAGAATATTGCAGACAGTCGTTCAGTACCTAATGCAATCATGGGGGAGAACGAGTAATTGGTAAATCACAAGGAAAGAAGTGTAGTCCACGGATGGACTCTCAAGGAGGTACAAGGTGCTATCATTAGACTTTAACAACGACTTGATTAAg                                            >  1:7011/1‑301 (MQ=255)`  
                                                                                                                                                                                                                                                                    `ttCCCAGGATCTCCGCTTCCGCAATATCTGGATTAAGTGTGCCAACAACTCTTGGAACTTCTTCCGTACTGGCCCCGATGGAATCTACTTCATAGCCTCTGATGGTGGATGGTTACGATTCCAAATACACTCCAACGGTCTCGGAGTCAAGAATATTGCAGACAGTCGTTCAGTACCTAATGCAATCATGGTGGAGAACGAGTAATTGGTAAATCACAAGGAAAGACGTGTAGTCCACGGATGGACTCTCAAGGAGGTACAAGGTGCTATCATTAGACTTTAACAACGAATTGATTAAGgc                                          <  1:2474/301‑1 (MQ=255)`  
                                                                                                                                                                                                                                                                            `aTCTCCGCTTCCGCAATATCTGGATTAAGTGTGCCAACAACTCTTGGAACTTCTTCCGTACTGGCCCCGATGGAATCTACTTCATAGCCTCTGATGGTGGATGGTTACGATTCCAAATACCCTCCAACGGTCTCGGATTCAAGAATATTGCAGACAGTCGTTCAGTACCTAATGCAATCATGGTGGAGAACGAGTAATTGGTAAATCAAAAGGAAAGCCGTGTAGTCCACGGATGGACTCTCAAGGAGGTACAAGGTGCTATCATTAGACTTTAACAACGAATGGATTAAGGCTGCTCaaa                                  >  1:465112/1‑301 (MQ=255)`  
                                                                                                                                                                                                                                                                               `tcCGATTCCGCAATAGCTGGATTAAGTGTACCAACAACTCTTGGAACTTCTTCCGTACTGGCCCCGATGGAATCTACTTCATAGCCTCTGATGGTGGATGGTTACGATTCCAAATACACTCCAACGGTCTCGGATTCAAGAATATTGCAGACAGTCGTTCAGTACCTAATGCAATCATGGTGGAGAACGAGTAATTGGTAAATCACAAGGAAAGACGTGTAGTCCACGGATGGACTCTCAAGGAGGTACAAGGTGCTATCATTAGACTTTAACAACGAATTGATTAAGGCTGCTCCAAttg                               <  1:6396/301‑1 (MQ=255)`  
                                                                                                                                                                                                                                                                                             `atCTGGATTAAGTGTACCAACAACTCTTGGAACTTCTTCCGTACTGGCCCCGATGGAATCTACTTCATAGCCTCTGATGGTGGATGGTTACGATTCCAAATACACTCCAACGGTCTCGGATTCAAGAATATTGCAGACAGTCGTTCAGTACCTAATGCAATAATGGTGGAGAACGAGTAATTGGTAAATCAACAGGAAAGACGTGTAGTCCACGGATGGACTCTCAAGGAGGTACAAGGTGCTATCATTAGACTTTAACAACGAATTGATTAAGGATGCTCCAATTGTTGGGACGGGTGTa                 >  1:53782/1‑301 (MQ=255)`  
                                                                                                                                                                                                                                                                                               `cttgATTAAGTGTGCCAACAACTATTGGAACTTCTTCCGTACTGGCCCGGATGGAATCTACTTAATAGCCTCTGATGGTGGATGGTTACGATTCCAAATACAATCCAACGGTCTCGGATTCAAGAATATTGCAGACAGGCGTTCAGCACCTAATGCAATCATGGTGGAGAACGAGTAATTGGTAAATCACAAGGAAAGACGTGTAGTCCACGGATGGACTCTCAAGGAGGTACAAGGTGCTATCATTAGACTTTAACAACGAATTGATTAAGGCTGCTCCAATTGTTGGGACGGGTGTAGc               <  1:351156/298‑1 (MQ=255)`  
                                                                                                                                                                                                                                                                                                 `ggATTAAGTGTGCCAACAACTCTTGGAACTTCTTCCGTACTGGCCCCGATGGAATCTACTTCATAGCCTCTGATGGTGGATGGTTACGATTACAAATACACTCCAACGGTCTCGGATTCAAGAATATTGCAGACAGTCGTTCAGTACCTAATGCAATCATGGTGGAGAACGAGTAATTGGTAAATCACAAGGAAAGACGTGTAGTCCACGGATGGACTCTCAAGGAGGTACAAGGTGCTCTCATTAGACATTAACAACGACTTGATTAAGGCTGCGACAATTGTTGGGACGGGTGTAGCAg             >  1:544267/1‑301 (MQ=255)`  
                                                                                                                                                                                                                                                                                                 `agaatAAGTGTACCAACAACTCTTGGAACTTCTTCCGTACTGGCCCCGATGGAATCTACTTCATAGCCTCTGATGGTGGATGGTTACGATTCCAAATACACTCCAACGGTCTCGGATTCAAGAATATTGCAGACAGTCGTTCAGTACCTAATGCAATCATGGTGGAGAACGAGTAATTGGTAAATCACAAGGAAAGACGTGTAGTCCACGGATGGACTCTCAAGGAGGTACAAGGTGCTATCATTAGACTTTAACAACGAATTGATTAAGGCTGCTCCAATTGTTGGGACGGGAGTAGCAg             <  1:8798/297‑1 (MQ=255)`  
                                                                                                                                                                                                                                                                                                  `gATTAAGTGTACCAACAACTCTTGGAACTTCTTCCGTACTGGCACCGATGGACTCTACTTCCTAGCCTCTGATGGTGGATGGTTACGATTTCAAATACACTCCAACGGTCTCGGATTCAAGAATATTGCAGACAGTCGCTCAGTACCTAATGCAATAATGGCGGAGAACGAGTAATTGGGAAATGACAAGGAAAGACGTGTAGTCCACGGATGGAACCTCATGGAGGTACAAGGTGCGATCATTAGACTTTATCATCGAATTGATAAAGGCCGTTCCAATTGTTGGGACGGATGTAGcctg            >  1:199181/1‑298 (MQ=255)`  
                                                                                                                                                                                                                                                                                                    `ttAAGTGTACCAACAACTATTGGAACTTCTTCCGTACTGGCCCCGATGGAATCTACTTCATAGCCTCTGATGGTGGATGGTTACGATTCCAAATACACTCCAACGGTCTCGGATTCAAGAATATTGCAGACAGTCGTTCAGTACCTAATGCAATCATGGTGGAGAACGAGTAATTGGTAAATCACAAGGAAAGACGGGTAGTCCACGGATGGACTCTCAAGGAGGTACAAGGTGCTATCATTAGACTTTAACAACGAATTGATTAAGGCTGCTCCAATTGTTGGGACGGGTGTAGCAGATg          <  1:215535/301‑1 (MQ=255)`  
                                                                                                                                                                                                                                                                                                        `gtgtGCCAACAACTCTTGGAACTTCTTCCGTACTGGCCCCGATGGAATCTACTTCATAGCCTCTGATGGTGGATGGTTACGATTCCAAATACACTCCAACGGTCTCGGATTCAAGAATATTGCAGACAGTCGTTCAGTACCTAATGCAATCATGGTGGAGAACGAGTAATTGGTAAATCACAAGGAAAGACGTGTAGTCCACGGATGGACTCTCCAGGAGGTACAAGGTGCTATCATTAGACTTTAACAACGAATTGATTAAGGCTGCTCCAATTGTTGGGACGGGTGTAGCCGCTGTTAg      >  1:75392/1‑301 (MQ=255)`  

                                                                                                                                                                                                                                                                                                            `|`                                                                                                                                                                                                                                                                                                              
`GAGGGTCAGATAACAACAATGACTGTACCTTCCACTCCTATGTACATGGTACGACCTTAACACTCAAGCAGGACTATGCAGTAGTTAACAAACACTTCCACGTAGGTCAGGCCGTTGTGGCCACTGATGGTAATATTCAAGGTACTAAGTGGGGAGGTAAATGGCTGGATGCTTACCTACGTG‑ACAGCTTCGTTGCGAAGTCCAAGGCGTGGACTCAGGTGTGGTCTGGTAGTGCTGGCGGTGGGGTAAGTGTGACTGTTTCACAGGATCTCCGCTTCCGCAATATCTGGATTAAGTGTGCCAACAACTCTTGGAACTTCTTCCGTACTGGCCCCGATGGAATCTACTTCATAGCCTCTGATGGTGGATGGTTACGATTCCAAATACACTCCAACGGTCTCGGATTCAAGAATATTGCAGACAGTCGTTCAGTACCTAATGCAATCATGGTGGAGAACGAGTAATTGGTAAATCACAAGGAAAGACGTGTAGTCCACGGATGGACTCTCAAGGAGGTACAAGGTGCTATCATTAGACTTTAACAACGAATTGATTAAGGCTGCTCCAATTGTTGGGACGGGTGTAGCAGATGTTAGTGCT  >  NC_001604.1/35822‑36421`

  

Alignment Legend

Aligned base mismatch/match (shaded by quality score): `ATCG/ATCG` `< 3 ≤` `ATCG/ATCG` `< 10 ≤` `ATCG/ATCG` `< 22 ≤` `ATCG/ATCG` `< 34 ≤` `ATCG/ATCG` `< 38 ≤` `ATCG/ATCG`

Unaligned base: `atcg`    Masked matching base: `atcg`    Alignment gap: `‑`    Deleted base: `‑`
